# Gromita: A Fully Integrated Graphical User Interface to Gromacs 4

**DOI:** 10.4137/bbi.s3207

**Published:** 2009-09-07

**Authors:** Diamantis Sellis, Dimitrios Vlachakis, Metaxia Vlassi

**Affiliations:** Institute of Biology, National Centre for Scientific Research “Demokritos”, 15310 Ag. Paraskevi Attikis, Greece. Email: meta@bio.demokritos.gr

**Keywords:** molecular dynamics, simulation, graphical user interface, gromacs, cross-platform

## Abstract

Gromita is a fully integrated and efficient graphical user interface (GUI) to the recently updated molecular dynamics suite Gromacs, version 4. Gromita is a cross-platform, perl/tcl-tk based, interactive front end designed to break the command line barrier and introduce a new user-friendly environment to run molecular dynamics simulations through Gromacs. Our GUI features a novel workflow interface that guides the user through each logical step of the molecular dynamics setup process, making it accessible to both advanced and novice users. This tool provides a seamless interface to the Gromacs package, while providing enhanced functionality by speeding up and simplifying the task of setting up molecular dynamics simulations of biological systems. Gromita can be freely downloaded from http://bio.demokritos.gr/gromita/.

## Introduction

With the ever growing need of studying complex biological systems in less time, the use of computers in biology is vital. The science of the *in silico* study of the motion of a biological system and its development over time is called Molecular Dynamics (MD). Molecular dynamics simulations provide a powerful tool to understand protein function (for a review see).^1^ Using computers to simulate biological systems started back in early 1980s. Back then the biological systems and their parameterization was very poor, due to limited software and processing power available. A decade later, it was possible to use macromolecular systems of complex proteins, rather than small compounds, and for the first time ever use of a solvent, rather than *in vacuo* simulations.^2^ Better understanding of protein structure along with the geometrical increase of computer processing power led to simulating huge protein systems for timescales longer that the 1 ns barrier. Eventually, useful insights into many biological systems have been gained by lengthy, more exhausting MD simulations.^3^,^4^

One of the fastest and most widely used MD suites available today is Gromacs.^5^,^6^ Gromacs is used mostly for large protein systems or membrane dynamics, since its optimized code provides fast and reliable calculations even on workstation computers. Recently, a new faster and improved version of Gromacs (v. 4) has been released.^7^ Gromacs can efficiently exploit single processors and achieve high performance through optimizing existing algorithms and novel faster routines. The most interesting part though is that it is also highly efficient at scaling up on parallel workstations. It is the only MD piece of software available today that encompasses an optimized and faster intra-communication decomposition algorithm as well as a parallel constraint solver.

Although there has been a variety of GUIs for Gromacs,^8^ none is compatible with the recently released and improved version 4.

In this work we present Gromita, a graphical user interface (GUI) to Gromacs latest version (v. 4). Gromita stands out by being the only GUI that is fully compatible with all versions of Gromacs available today, as well as being the only real flexible cross-platform GUI capable of running virtually on every machine running Gromacs. Gromita by combining all Gromacs’s algorithms provides an efficient, fast and easy-to-use alternative MD suite that outperforms rather expensive rival MD suites.^6^,^9^

### Description of the program

Gromita is a cross-platform, perl/tcl-tk based, interactive front end to the latest update of Gromacs suite (ver. 4). Gromacs remains a native set of numerous UNIX-based modules, lacking a graphical user interface (GUI). Therefore, the Gromita GUI was developed to ease and automate the task of setting up molecular dynamics simulations with Gromacs in UNIX-shell native mode, a process that may prove to be time-consuming and error-prone for some users.

**Figure 1.**
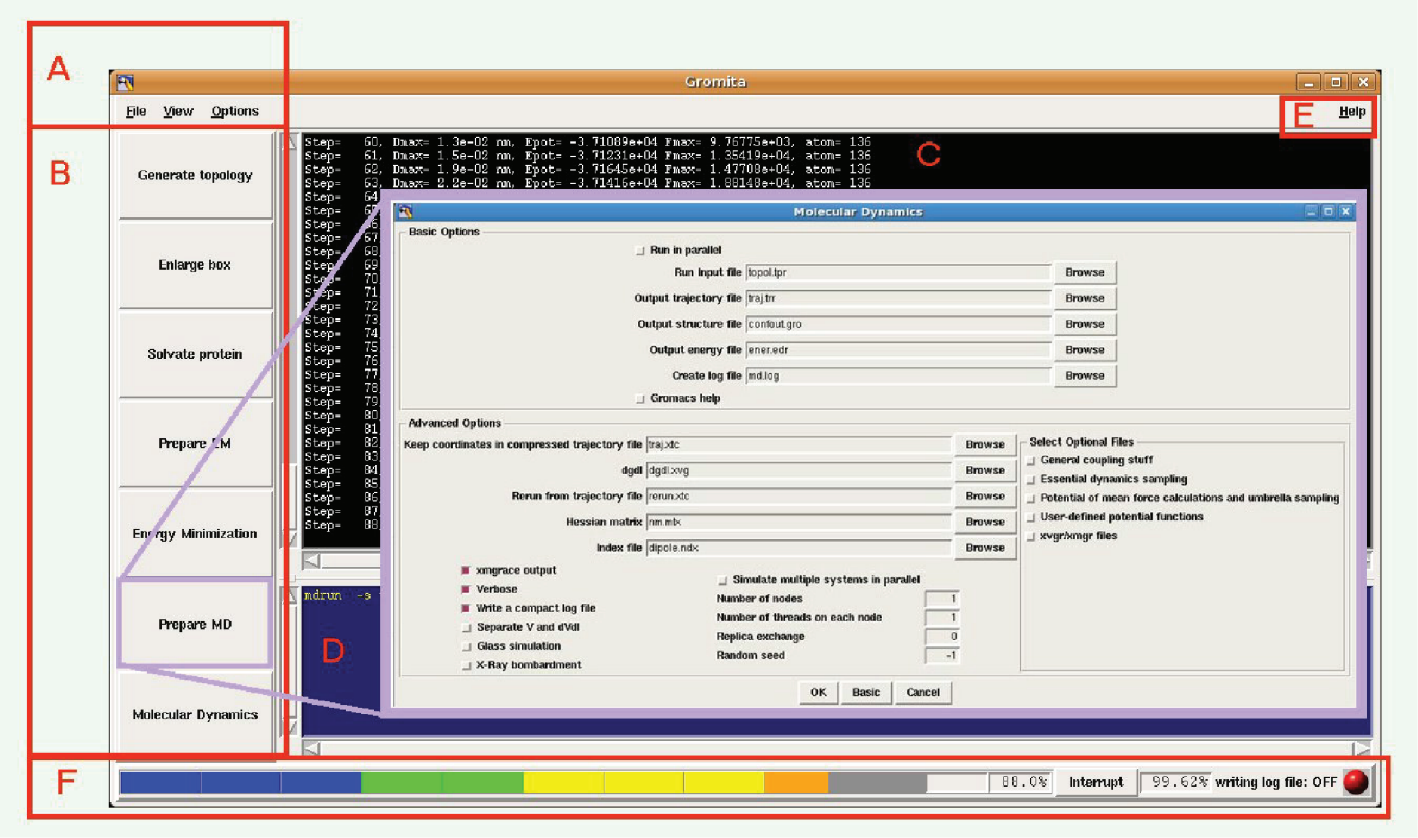
**The main window of the Gromita GUI. A**) the menu bar, **B**) the push-button MDs setup area, **C**) the Gromacs real-time interactive monitor, **D**) the command line translator window, **E**) the Help access button and **F**) the system status tray area. ***Insert***: The molecular dynamics preparation window. All options have been included in clickable buttons, switches or drop-down menus.

Gromita’s main-window is a menu-driven interface (Fig. 1A) as well as a button step-by-step layout (Fig. 1B). In addition, Gromita provides the user with a process window (Fig. 1C) to monitor Gromacs calculations in real time as well as with a command-line equivalent for all the GUI use (Fig. 1D). An extensive manual for the use of Gromita, will pop-up through the Help button (Fig. 1E), using the system’s default HTML browser. Finally, the system status tray area (Fig. 1F) provides information about the logging process plus a process interruption switch. Moreover, the progress indicator bar will emerge when the verbose option from the energy minimization and molecular dynamics push-buttons is selected. There are three progress indicators, a progress bar showing the current step as a fraction of the total steps, a label showing an estimation of the remaining run time and the percentage (%) of work completed (Fig. 1F). Calculations and step counts are done by Gromacs.

Furthermore, through the Gromita interface is possible to start a log file using the [File>log] module, an option lacking in Gromacs. The status of logging appears in the right/bottom of the main window in the form of a green or red indicator, for ease of use. Logging Gromac’s shell output is vital for keeping track of all useful information. A CPU percentage gauge is to be found right next to the logging indicator, providing real time information of the CPU load.

The full process for setting up and running a complete Gromacs molecular dynamics simulation has been incorporated to the Gromita GUI with full parameterization. First, the biological system has to be loaded through the menu bar. Thereon, the process is broken down in seven steps:

1. Generate topology: The Generate topology button opens a window enabling the user to add hydrogens, create coordinates (in Gromacs (Gromos) format) and topology files (in Gromacs format) from the coordinate file loaded. This button corresponds to the Gromacs command ‘pdb2gmx’. The basic options include a force field selector and the filenames of the coordinates and topology files, whereas the advanced options allow for full parameterization for this step.
2. Enlarge box: In this step a box of water with custom shape and size is defined or modified. It corresponds to the Gromacs command ‘editconf’, which converts generic structure format to Gromacs readable format (*.gro, *.g96 or *.pdb). It can also rotate coordinates and velocities as well as align the principal axes of the system along the coordinate axes, which may allow decreasing the system box volume.
3. Solvate protein: Solvates a molecule in a box of water molecules, determines the solute configuration and inserts a number of extra user-defined molecules, if required. It corresponds to the Gromacs command ‘genbox’.
4. Prepare EM (Energy Minimization): Corresponds to the Gromacs preprocessor ‘grompp’. In this step the binary files for the energy minimization step are prepared.
5. Energy Minimization: In this step the actual energy minimization process is performed and corresponds to the Gromacs command ‘mdrun’. Gromita takes full advantage of all parameters of ‘mdrun’ as set within Gromacs. ‘Mdrun’ program is the main computational chemistry engine within Gromacs. It either performs conjugate gradient or steepest descent energy minimization. Normal mode analysis is another capability of ‘mdrun’. In this case the algorithm builds a Hessian matrix, starting from a single conformation. For most normal mode-like calculations and simulations, the structure provided has to be properly energy-minimized.
6. Prepare MD (Molecular Dynamics): This step corresponds to the Gromacs preprocessor ‘grompp’. In this step the binary files for the molecular dynamics simulation are prepared. The basic and advanced options window of this step is shown in Figure. 1, *Insert.*
7. Molecular Dynamics: In this step the actual molecular dynamics simulation process is performed through the Gromacs command ‘mdrun’. Brownian or Langevin dynamics may also be performed at this step.

Post MD simulation trajectory analysis has not been incorporated into the Gromita GUI for two main reasons: Firstly, a wide range of the most basic and important analysis tools have already been included in the Gromacs 4 suite, which also includes the Gromacs-native trajectory viewer. Secondly, quite recently a freeware molecular dynamics trajectory analysis tool has been released^9^ that is fully compatible with Gromacs.

In conclusion, the Gromita GUI provides a novel, user-friendly, fast and reliable cross-platform tool for conducting molecular dynamics simulations with all versions of Gromacs. The Gromita GUI provides an efficient, fast and easy-to-use alternative to all available MD software packages, including rather expensive commercial suites, whilst being distributed as freeware. Most importantly, Gromita is the only GUI existing today compatible with the latest version of Gromacs (version 4).

## References

[1] Karplus M Kuriyan Molecular Dynamics and Protein Function J Proc Natl Acad Sci U S A 2005 102 19 6679 85 10.1073/pnas.0408930102 PMC1100762 15870208

[2] van Gunsteren WF Bakowies D Baron R Biomolecular Modeling: Goals, Problems, Perspectives Angew Chem Int Ed Engl 2006 45 25 4064 92 16761306 10.1002/anie.200502655

[3] Deliot N Chavent M Nourry C Biochemical Studies and Molecular Dynamics Simulations of Smad3-Erbin Interaction Identify a Non-Classical Erbin PDZ Binding Biochem Biophys Res Commun 2009 378 3 360 5 19013433 10.1016/j.bbrc.2008.10.175

[4] Peters GH The Effect of Asp54 Phosphorylation on the Energetics and Dynamics in the Response Regulator Protein Spo0F Studied by Molecular Dynamics Proteins 2009 75 3 648 58 19004019 10.1002/prot.22276

[5] Lindahl E Hess B van der Spoel D GROMACS 3.0: A Package for Molecular Simulation and Trajectory Analysis J Mol Model 2001 7 8 306 17

[6] Van der Spoel D Lindahl E Hess B Groenhof G Mark AE Berendsen HJ GROMACS: Fast, Flexible, and Free J Comput Chem 2005 26 16 1701 18 16211538 10.1002/jcc.20291

[7] Hess B Kutzner C van der Spoel D Lindahl E GROMACS 4: Algorithms for Highly Efficient, Load-Balanced, and Scalable Molecular Simulation J Chem Theory Comput 2008 4 435 47 10.1021/ct700301q 26620784

[8] http://www.gromacs.org/WIKI-import/GUI

[9] Kutzner C van der Spoel D Fechner M Speeding Up Parallel GROMACS on High-Latency Networks J Comput Chem 2007 28 12 2075 84 17405124 10.1002/jcc.20703

[10] Glykos NM Carma: a Molecular Dynamics Analysis Program J Comput Chem 2006 27 14 1765 8 16917862 10.1002/jcc.20482

